# Biomarkers for Prostate Cancer Aggressiveness in Puerto Rican Men: Analysis of Phospho-Rb S249, N-cadherin, β-catenin, and E-cadherin Expression in Prostate Biopsies

**DOI:** 10.71332/wdwgem70

**Published:** 2025-07-14

**Authors:** Sheila M. Valle Cortés, Jaileene Pérez Morales, Mariely Nieves Plaza, Raymond Quiñones Alvarado, Gilberto Ruiz Deyá, Juan C. Santa Rosario, Pedro Santiago Cardona

**Affiliations:** 1 Ponce Research Institute, Ponce Health Sciences University, Biochemistry and Cancer Biology Divisions, Ponce, PR; 2 Knight Cancer Institute, Oregon Health & Science University, Department of Population Sciences, Portland, US; 3 Universidad Central del Caribe, Bayamón, PR; 4 University of Puerto Rico, Mayagüez, PR; 5 CorePlus Servicios Clínicos y Patológicos, Carolina, PR

**Keywords:** Prostate cancer, β-catenin, Puerto Rico, Epithelial-to-Mesenchymal Transition (EMT)

## Abstract

Prostate cancer (PCa) is the leading cause of cancer in Puerto Rican men and exhibits significant racial disparities globally. Although only 8% of cases invade beyond the prostate, predicting PCa aggressiveness is challenging. This study investigated the potential of the retinoblastoma tumor suppressor protein phosphorylated in Serine 249 (Phospho-Rb S249), N-cadherin, β-catenin, and E-cadherin as biomarkers for identifying aggressive PCa in Puerto Rican men. We hypothesized that the expression of these proteins could serve as biomarkers for identifying PCa tumors with potential to becoming aggressive in Puerto Rican men. Immunohistochemistry was performed on 23 biopsies from Puerto Rican men to evaluate the biomarkers’ expression, and correlation analyses examined associations with clinicopathological parameters. Results showed that Phospho-Rb S249 expression correlated positively with tumor size and positive cores in patients with Gleason scores ≥4+3. β-catenin was positively associated with tumor size and carcinoma percentage in Gleason scores ≥4+3. E-cadherin expression negatively correlated with grade group, indicating a protective role. In contrast, N-cadherin and β-catenin were more prominent in Gleason scores ≤3+4, hinting at their involvement in early epithelial-to-mesenchymal transition (EMT). A decision tree analysis identified N-cadherin expression as a key determinant for classifying PCa aggressiveness, with an 82% likelihood. These findings suggest N-cadherin as a biomarker for identifying PCa with the potential to become aggressive. While our study provides promising results, further validation in a larger patient cohort is needed to increase the robustness and reliability of our findings. Also, combining multiple biomarkers could further enhance the specificity of aggressive PCa detection.

## 1. Introduction

Prostate cancer (PCa) accounted for 29% of all cancer diagnoses in 2023, and since 2010, there has been an increase in cases diagnosed at higher grades and advanced stages [1,2]. For PCa to metastasize, cancer cells must acquire a migratory and invasive phenotype [3]. However, only about 8% of cases invade beyond the prostate to other body parts like the bones or lymph nodes [4]. Thus, overtreatment is a common problem in PCa screening, ranging from 1.7% to 67% of cases [5]. PCa overtreatment leads to long-term urinary, erectile, and bowel dysfunction in prostatectomy and radiation patients, impacting the patient’s quality of life and healthcare costs [6]. Thus, prognostic tests, including gene panels like Prolaris and Oncotype DX, have been developed to help clinicians identify PCa with aggressive potential and guide disease management by predicting the likelihood of progression [7]. However, evidence supporting their clinical utility is limited. Prolaris showed no significant impact on treatment plans or patient outcomes, while Oncotype DX had mixed effects on physician treatment recommendations [8,9,10]. Moreover, the effectiveness of Oncotype DX is diminished in African American men because the test mainly relies on data from Caucasian populations, limiting its relevance for other ethnic groups [11].

Minorities, such as Black and Hispanic men, are underrepresented in PCa clinical trials, making it challenging to manage their disease [12]. Compared to US-Hispanics and non-Hispanic whites, Puerto Ricans have a 40% higher PCa mortality rate, associated with low socioeconomic status [13]. Additionally, studies evaluating Hispanic/Latino populations have found that Puerto Ricans experience higher (HR=1.70, 95% CI 1.28 to 2.25, *p*<0.001) PCa-specific mortality compared to other Hispanic/Latino groups [14]. This heterogeneity affects clinical practice, as minority groups are often categorized in US or Hispanic data, which may not reflect the true impact of the disease in these populations [15]. Therefore, we cannot be certain whether existing PCa prognostic tests will be equally effective in Puerto Ricans.

Cadherin and catenins are crucial for epithelial cell adhesion, and their disruption can promote tumor progression [16]. E-cadherin has been shown to play a crucial role in maintaining the integrity of adherens junctions and epithelial organization [17]. During embryonic development, E-cadherin is crucial for tissue formation and cell rearrangement, while its reduced expression in adult tissues is associated with loss of contact inhibition, increased cell motility, and advanced cancer stages [18,19]. β-Catenin interacts with other catenins and plays an anti-oncogenic role when localized at the cell membrane [20]. However, when overexpressed in the nucleus of cells, it becomes oncogenic and promotes invasive PCa [20]. The reduced expression or functional loss of E-cadherin and its complex with β-catenin is considered a hallmark of epithelial-to-mesenchymal transition (EMT), a process in which epithelial cells change into mesenchymal cells, leading to cancer spread [21,22]. During EMT, a cadherin switch from E-cadherin to N-cadherin occurs, which strongly suggests PCa progression and poor prognosis [23]. This emphasizes the importance of E-cadherin, N-cadherin, and β-catenin as potential biomarkers for detecting aggressive PCa and monitoring disease progression.

We studied the Retinoblastoma (Rb) protein, a critical regulator of the cell cycle, for its potential in the early detection of aggressive PCa in Puerto Rican men. Loss of Rb’s tumor suppressive function can induce cell cycle deregulation and lead to a malignant phenotype in various cancers [24]. Post-translational modifications, such as phosphorylation, on Rb play a crucial role in modulating its function and cancer progression [25]. In PCa, Rb loss occurs during disease progression, particularly as tumors become resistant to castration treatments [26]. However, there is a lack of studies examining the functional role of Rb phosphorylation sites. In our previous research on lung cancer, we found a positive correlation between Phospho-Rb S249 levels and tumor grade, indicating a potential link between this specific phosphorylation and tumor aggressiveness [27].

In a previous PCa study conducted in our laboratory, these biomarkers were assessed in an Asian cohort. The findings indicated that in Asian patients, there is a negative correlation between the expression of E-cadherin and β-catenin with aggressive tumor behavior, whereas Phospho-Rb S249 and N-cadherin were positively correlated with increased tumor aggressiveness [28]. Furthermore, when Asian patients were stratified based on Gleason scores to assess the ability of the biomarkers to identify aggressive PCa, β-catenin emerged as a key classifier for distinguishing between low- and high-risk diseases [28]. Thus, in this study, we assessed the potential of Phospho-Rb S249, N-cadherin, β-catenin, and E-cadherin as biomarkers for identifying PCa tumors with a high risk of becoming aggressive among Puerto Rican men. We hypothesized that the expression levels of these biomarkers could help distinguish PCa tumors with aggressive potential in this population. Our results showed that in patients with Gleason score ≤3+4, E-cadherin negatively correlated with tumor’s grade group. In contrast, in patients with Gleason scores ≥4+3, Phospho-Rb S249 was positively correlated with tumor size and the total number of positive cores. Additionally, β-catenin was positively correlated with tumor size and the percentage of carcinoma in the tissue, while E-cadherin was negatively correlated with tumor’s grade group. Also, N-cadherin and β-catenin were more prominent in patients with Gleason scores ≤3+4, potentially due to their involvement in early EMT. Finally, a classification tree revealed that N-cadherin expression is a critical biomarker for identifying Puerto Rican PCa patients with a high potential for aggressive disease, indicating that this biomarker may have practical utility in clinical settings.

## 2. Methodology

### 2.1 Puerto Rican PCa biopsy samples

Puerto Rican PCa biopsy samples were obtained from “CorePlus Servicios Clínicos y Patológicos” in Carolina, Puerto Rico. In this study, all patient biopsy samples were retrospectively collected; we did not perform any patient recruitment. Thus, we received an Institutional Review Board (IRB) exemption with protocol number 2207109755. The samples provided by “CorePlus Servicios Clínicos y Patológicos” were already prepared as formalin-fixed and paraffin-embedded on a positively charged glass slide. Each slide contained a single patient biopsy sample, with each tumor section measuring 5μm in thickness. Also, “CorePlus Servicios Clínicos y Patológicos” provided the demographic and pathological information for each patient. The information included the patient’s age, PSA levels (ng/ml), tumor size (millimeters), grade group, Gleason score, percentage of the biopsy with carcinoma, and the number of positive cores for each patient. The control group consisted of prostate biopsy samples selected based on the absence of carcinoma, classified as negative following standard pathological evaluation. In total, 23 PCa adenocarcinoma cases and nine prostate control cases were analyzed in the study.

### 2.2 Immunohistochemistry (IHC)

Immunohistochemistry (IHC) was performed to assess the expression of Phospho-Rb S249, N-cadherin, β-catenin, and E-cadherin in the PCa biopsy samples, following a previously established protocol [29]. Paraffin was first removed from the slides, and the samples were hydrated and treated to block endogenous peroxidase activity. Antigen retrieval was then carried out, followed by incubation with primary and secondary antibodies. The antibodies used in this study were Phospho-Rb S249 (Abcam, Cat. No. ab4788) 1:100, purified mouse anti-N-cadherin (BD Biosciences, Cat. No.610920) 1:125; β-catenin (Cell Signaling, Cat. No. 8480S) 1:200; and E-cadherin (Cell Signaling, Cat. No. 3195S) 1:400. The secondary antibody used was the Super Sensitive Link Label IHC kit (BioGenex, Cat. No. LP000-ULE). All samples were exposed to one drop of Diaminobenzidine (DAB) (BioGenex, Cat. No. HK542-XAKE) for one minute and 30 seconds. Then, slides are dehydrated, cleared in xylene, and mounted with coverslips, to be analyzed under a light microscope. Slides were preserved with a coverslip and Cytoseal 60 (Thermo Scientific, Cat. No. 8310-16) to protect the samples and ensure optimal examination on the microscope for the image acquisition. The image acquisition was performed using the NIS-Element AR software and the Olympus BX60 microscope. The expression of N-cadherin, β-catenin, and E-cadherin was assessed based on their membranous localization, while Phospho-Rb S249 expression was evaluated in the nucleus. This is because N-cadherin, β-catenin, and E-cadherin function as epithelial markers, whereas when Rb gets phosphorylated and inactivated, it exhibits nuclear localization. All tissue cores were analyzed in four quadrants at 40X magnification. Immuno-stained slides were independently and blindly scored for the number of immune-positive cells in each biopsy. We assigned scores using objective measures with the Image J program, which automatically provided a scale of positivity after selecting a manual threshold of 37%. This scale was categorized as negative, low positive, positive, or high positive. We translated this scale into a numerical format ranging from 1 to 4, with 1 indicating a negative result and 2, 3, and 4 representing low positive, positive, and high positive results, respectively.

### 2.3 Statistical Analysis

Graph Pad Prism software version 9 was used to describe the sample population using measures of central tendency and dispersion (mean, standard deviation, median, 25^th^ and 75^th^ percentiles) for continuous variable; categorical variables were described with frequencies and percentages. A Spearman correlation analysis was performed to assess the strength and direction of the relationship between the biomarker’s expression and the clinicopathologic data. Patients were stratified into two groups based on their Gleason score, following the National Comprehensive Cancer Network (NCCN) guidelines (Table 1) [30]. One group consisted of patients with a Gleason score pattern equal to or less than 3+4, whereas the second group consisted of patients with a Gleason score pattern equal to or higher than 4+3. The Wilcoxon rank-sum test was employed to perform group comparisons after stratifying patients based on their Gleason score. This analysis was performed using STATA version 17 (STATA Corp, College Station, TX, USA). Lastly, R Studio version 4.3.0 was used to construct a classification tree aimed at assessing whether the expression of the biomarkers could identify patients at risk of developing aggressive PCa. A split ratio of 0.70 was employed to allocate a larger portion of the dataset to the training set, enabling better tree construction. Additionally, parameters were set with a minimum split of five and a complexity parameter (cp) value of 0.01 to control tree depth and complexity. We also assessed the performance of the predictive model by analyzing its Receiver Operating Characteristic (ROC) curve. A significance level of *p*≤0.05 was established to be statistically significant for all analyses.

## 3. Results

### 3.1 Biomarker expression and Gleason score stratification in Puerto Rican PCa patients

**Figure 1.**
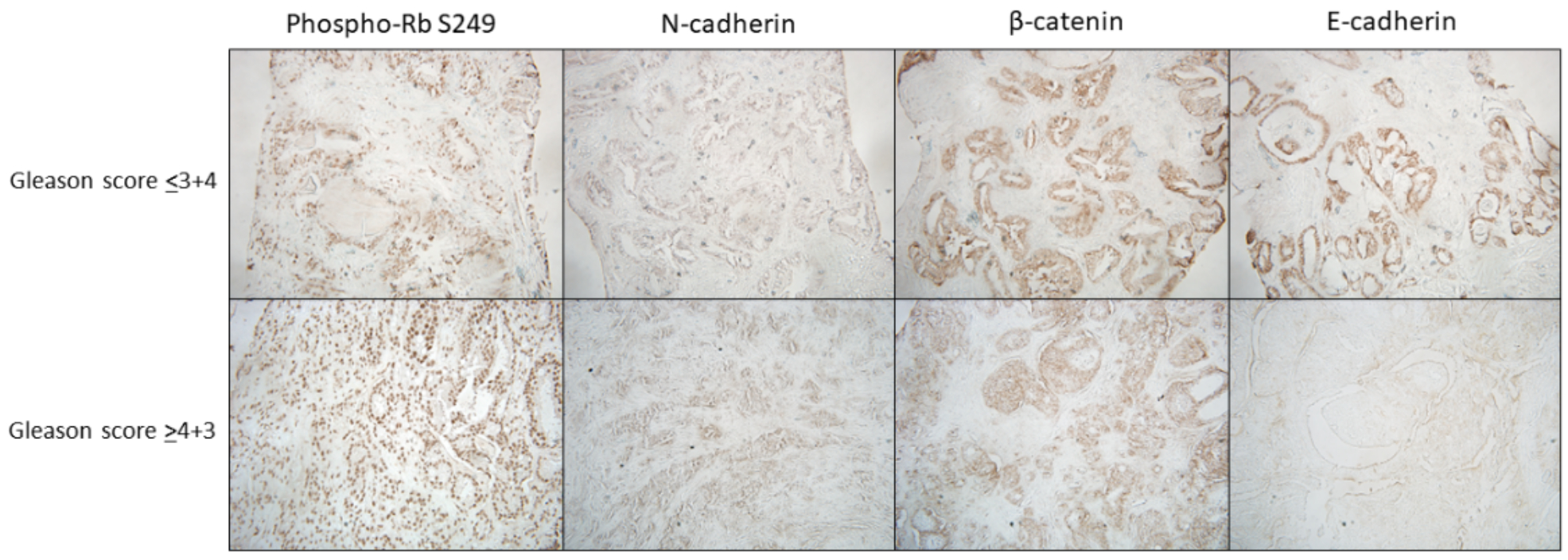

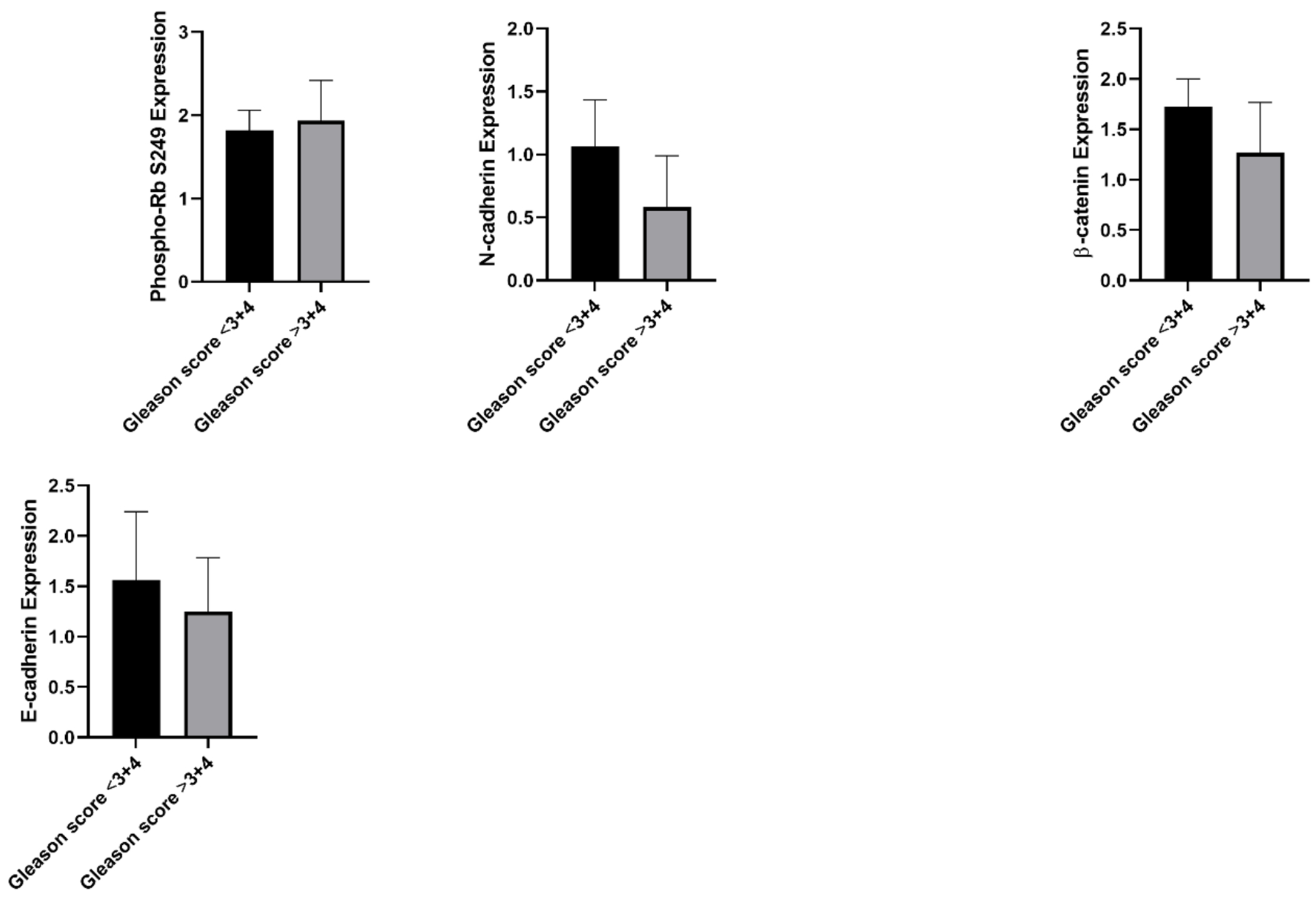
IHC staining of the biomarkers Phospho-Rb S249, N-cadherin, β-catenin, and E-cadherin. The upper section of the figure corresponds to a patient with a Gleason score of 3+3, representing the staining patterns that predominate within the Gleason scores ≤3+4, a less aggressive form of PCa. In the lower section of the figure, the staining corresponds to a patient with Gleason score of 4+5, representing the pattern that predominates in patients with Gleason scores ≥4+3, a more aggressive form of PCa. Pictures were captured at 40X visual fields. Bars represent mean expression levels, and error bars indicate ± standard deviation (SD).

To investigate the potential of Phospho-Rb S249, N-cadherin, β-catenin, and E-cadherin as biomarkers for identifying aggressive PCa in Puerto Rican patients, we stratified the patients based on their Gleason score (N=23), a key factor in determining the prognosis of prostatic malignancies [31]. PCa patients with a Gleason score of 4+3 or higher have worse overall survival, cancer-specific survival, and increased risk of progression compared to those with Gleason score 3+4 or lower [32,33]. Accordingly, one group included patients with Gleason scores of 3+4 or lower (N=8), and the other included patients with Gleason scores of 4+3 or higher (N=15). Figure 1 illustrates the IHC staining results for the expression of the biomarkers (Phospho-Rb S249, N-cadherin, β-catenin, and E-cadherin) stratified by Gleason score categories (≤3+4 vs. ≥4+3) based on their mean expression levels. Nuclear expression of Phospho-Rb S249 was not significantly different between the groups (*p*-value: 0.06250). N-cadherin expression was slightly higher in patients with Gleason scores ≤3+4 compared to those with scores ≥4+3 (*p*-value: 0.0547). In contrast, β-catenin and E-cadherin expression levels were slightly higher in the ≤3+4 group than in the ≥4+3 group, with *p*-values of 0.0859 and 0.5625, respectively. However, no significant differences were observed in the expression levels of these biomarkers between the Gleason score categories.

Table 2 presents the frequency and percentage of clinicopathological parameters based on Gleason score. No differences were observed in age between the groups (*p*-value>0.05). The median tumor size was larger in patients with Gleason score ≥4+3 (6.75 mm vs. 5.25 mm), although this difference was not statistically significant (*p*-value>0.05). Similarly, patients with Gleason scores ≥4+3 had a higher median percentage of carcinoma in their biopsies (34.0% vs. 30.5%) and a greater median number of positive cores (6.0 vs. 2.0), with the latter nearing statistical significance (*p*-value: 0.054). We further examined how Phospho-Rb S249, N-cadherin, β-catenin, and E-cadherin were expressed across the Gleason scores categories based on the median (Table 3). N-cadherin expression was higher in patients with Gleason scores ≤3+4, with a median of 1.12 compared to 0.75 in those with Gleason scores ≥4+3 (*p*-value: 0.023). Also, β-catenin showed statistical significance in patients with Gleason scores ≤3+4 (median:1.75 vs. 1.25 for Gleason scores ≥4+3, *p*-value: 0.018). However, there were no significant differences in the expression of Phospho-Rb S249 or E-cadherin between the two Gleason score categories in this cohort (*p*-value>0.05).

### 3.2 Correlation analysis between biomarker expression and clinicopathological parameters

We conducted a correlation analysis to explore the relationships between the expression of Phospho-Rb S249, N-cadherin, β-catenin, and E-cadherin, and various clinical parameters, including age, PSA, grade group, tumor size, percentage of carcinoma, and the number of positive biopsy cores (Tables 4 and 5). In patients with Gleason scores ≤3+4, we observed a significant negative correlation between E-cadherin expression and the grade group (r: −0.759, *p*-value: 0.029), a prognostic scale ranging from one to five linked to biochemical recurrence and prostate-specific mortality [34]. However, no significant correlations were found for Phospho-Rb S249, N-cadherin, or β-catenin with any clinical parameter in this category. In contrast, Phospho-Rb S249 expression was positively correlated with tumor size (r: 0.616, *p*-value: 0.019) and the number of positive biopsy cores (r: 0.522, *p*-value: 0.046). Similarly, β-catenin expression showed positive correlations with tumor size (r: 0.570, *p*-value: 0.033) and the percentage of carcinoma in the biopsy samples (r: 0.635, *p*-value: 0.011). Additionally, E-cadherin negatively correlated with the grade group (r: −0.566, *p*-value: 0.028). No significant correlations were identified between N-cadherin expression and any clinicopathological parameters in patients with Gleason scores ≥4+3.

### 3.3 Classification tree analysis for aggressive PCa identification

**Figure 2.**
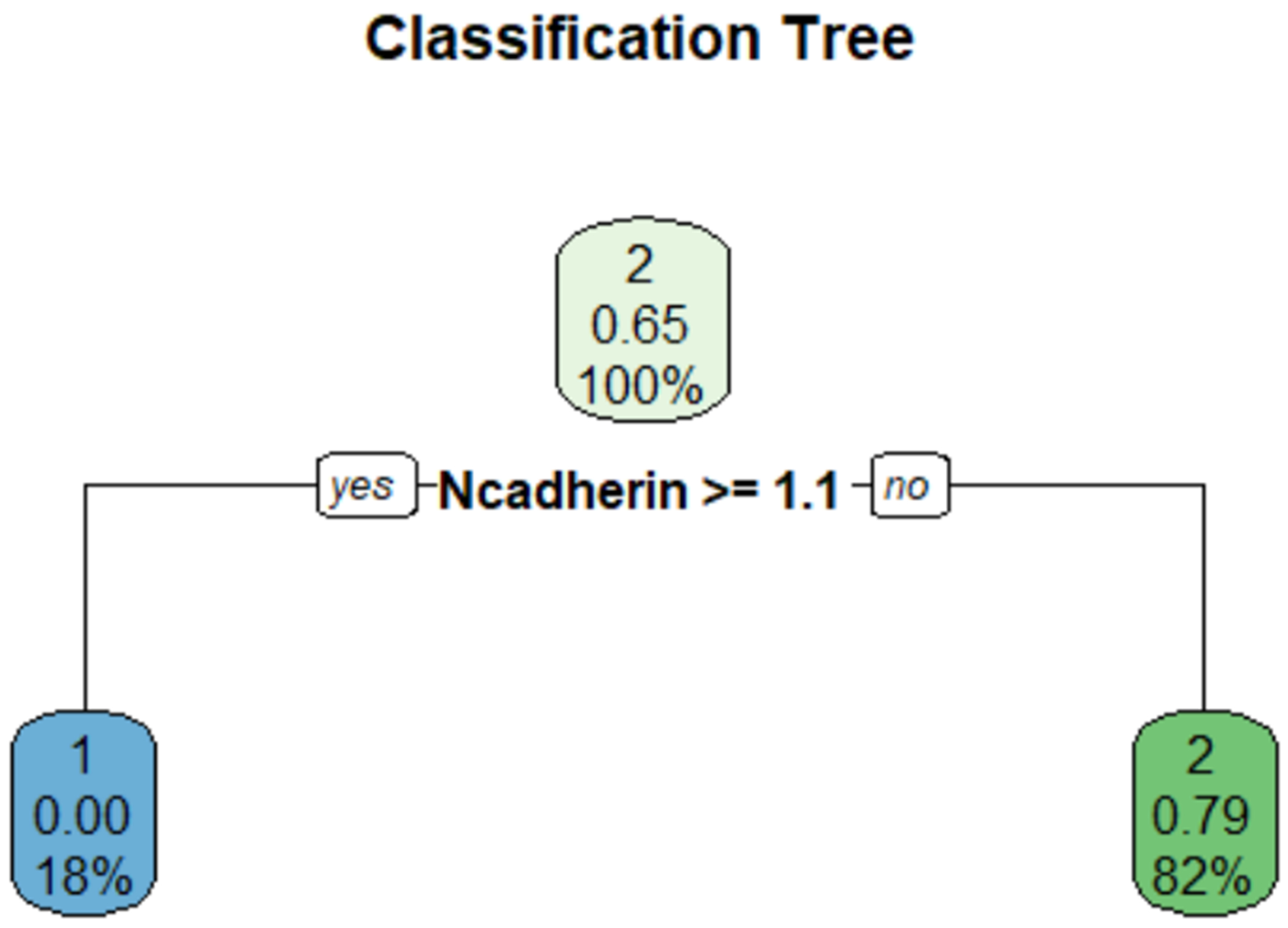
Classification tree for predicting PCa aggressiveness based on N-cadherin expression levels.

To evaluate whether the expression of the biomarkers Phospho-Rb S249, N-cadherin, β-catenin, and E-cadherin could identify patients at risk of developing an aggressive PCa, a classification tree was constructed (Figure 2). This analysis identified that N-cadherin expression is a critical determinant for classifying the PCa aggressiveness in this dataset. Specifically, patients with N-cadherin expression levels ≥1.1 (low positive or higher) have an 18% probability of having Gleason scores ≤3+4, with a predicted likelihood of 0% (0.00) belonging to the Gleason score ≥4+3 category. Conversely, PCa patients with N-cadherin expression levels <1.1 (negative expression) were classified as having Gleason scores ≥4+3, with an 82% probability and a 79% (0.79) predicted likelihood of belonging to it. The classification tree demonstrated an AUC of 0.75.

## 4. Discussion

This study aimed to evaluate the potential of Phospho-Rb S249, N-cadherin, β-catenin, and E-cadherin as biomarkers for identifying PCa with high risk of becoming aggressive in Puerto Rican men. For this, we explored the relationship between the expression of Phospho-Rb S249, N-cadherin, β-catenin, and E-cadherin with the clinicopathological parameters of the Puerto Rican PCa patients. When evaluating the clinicopathological parameters, we observed an overall trend of increasing tumor aggressiveness in patients with higher Gleason scores. Furthermore, the evaluation of the biomarker’s expression levels showed that N-cadherin and β-catenin were highly expressed in Gleason scores ≤3+4. These results potentially reflect the early onset of EMT, contributing to disease progression.

N-cadherin expression has been noted in multiple human cancers, such as those of the breast, lung, and liver, among others [35,36,37]. This occurs in some cases irrespective of E-cadherin status [38,39]. The appearance of N-cadherin expression is among the earliest events in malignant transformation. Also, low N-cadherin expression has been found in cervical lesions and neoplasia, that have precancerous potential [40,41]. Additionally, the hallmark of EMT starts with the upregulation of N-cadherin, accompanied by the downregulation of E-cadherin, a process regulated by a complex network of signaling pathways and transcription factors [42]. Consequently, the presence of N-cadherin in the early stages of cancer may stimulate cell migration and invasion, contributing to tumorigenesis [43].

The expression pattern of N-cadherin observed in this Puerto Rican cohort suggests that lower Gleason score tumors might exhibit elevated N-cadherin levels, possibly indicating a role in early tumor characteristics rather than advanced disease progression. Additionally, the high levels of β-catenin in patients with Gleason scores ≤3+4 emphasize its role in maintaining cell-cell adhesion during the initial stages of the disease. The interaction between β-catenin and E-cadherin at the membrane may prevent β-catenin from translocating to the nucleus and participating in the transcriptional activation of factors such as LEF-1/TCF [44]. Conversely, studies have shown that Wnt signaling activation can increase β-catenin levels in the membrane, enhancing its recruitment to existing adherens junctions [45]. Thus, the presence of N-cadherin and β-catenin in tumors with Gleason scores ≤3+4 may suggest an early transition toward more aggressive disease. Furthermore, our findings indicated that reduced E-cadherin expression was significantly associated with the grade group in patients with both Gleason scores ≤3+4 and ≥4+3. This association suggests that E-cadherin downregulation is closely related to more aggressive and poorly differentiated tumors. Consequently, decreased E-cadherin expression may be indicative of more aggressive disease in high-risk patients. Our findings align with previous studies that emphasize the role of E-cadherin dysfunction or loss of expression in cancer progression, as it leads to reduced cellular adhesion in epithelial tissues [18].

Moreover, we observed correlations between increased β-catenin expression with the percentage of carcinoma in biopsies and tumor size in patients with Gleason scores ≥4+3. It is well established that E-cadherin inhibits the nuclear localization and transactivation of β-catenin [46]. Therefore, the loss of E-cadherin’s growth suppressor activity disrupts this inhibition, allowing β-catenin to translocate into the nucleus, where it can bind to TCF/LEF and activate the Wnt/β-catenin signaling pathway, promoting tumorigenesis [47]. This finding is consistent with the established role of β-catenin in cancer progression, as its nuclear translocation is often associated with increased invasiveness and metastasis in various cancers [48,49,50]. In vitro studies have also confirmed that the disruption of β-catenin and E-cadherin leads to a loss of intercellular adhesion and enhanced tumor invasion in human cancer cells [51]. Consequently, our findings of increased β-catenin expression in patients with Gleason scores ≥4+3 may reflect the nuclear translocation of this protein and the activation of the Wnt/β-catenin signaling pathway. Importantly, while N-cadherin did not show significant correlations with the clinicopathological parameters in this cohort of Puerto Rican PCa patients, its role remains relevant, especially in the context of EMT.

We also assessed the potential of Phospho-Rb S249 as a biomarker for identifying PCa with potential to become aggressive. The Rb protein contains 14 mono-phosphorylated sites, each potentially influencing Rb interactions and conferring functional specificity [52]. Rb is known to be inactivated through hyper-phosphorylation, and distinct phosphorylation patterns are observed in different cancer types, suggesting that phosphorylation-based mechanisms may drive Rb dysfunction in a cancer-specific manner [53]. Thus, it has been suggested that Rb protein may have a phosphorylation code that controls specific activities in various processes regulating proliferation [53]. However, the role of Rb S249 has only been studied in conjunction with threonine 252 (T252) in previous research [54,55]. Notably, no clear association has been established between specific Rb phosphorylation events and PCa progression. While limited research exists on Phospho-Rb S249 in cancer, our previous study on lung cancer found that combined expression of Phospho-Rb S249 with p39 and E-cadherin was associated with advanced tumor staging in non-small cell lung carcinoma [27]. Here, we have identified that increasing levels of Phospho-Rb S249 correlated with tumor size and number of positive cores in Puerto Rican patients with Gleason scores ≥4+3, revealing a potential role in aggressive PCa. However, Phospho-Rb S249 has a role during early G1, where cyclin D/Cdk4/6 phosphorylates Rb at residues S249, T356, S807, S811, and T826, whereas in late G1 and early S, cyclin E/Cdk2 phosphorylates Rb at residues S612 and T821 [56]. Therefore, subsequent phosphorylation events, such as those at S612 or T821, in conjunction with S249 may provide better predictive value for identifying PCa patients at risk of progressing to aggressive disease; however, this hypothesis requires further investigation.

Comparing our findings with those of our previous study on Asian PCa patients, both studies demonstrated that Phospho-Rb S249, N-cadherin, β-catenin, and E-cadherin were significantly associated with tumor aggressiveness [28]. However, differences in biomarker behavior were observed between the populations. In the Asian cohort, β-catenin emerged as the primary classifier for aggressive PCa, whereas in the Puerto Rican cohort, N-cadherin was the most informative biomarker for distinguishing aggressive PCa. These findings are important when considering population-specific molecular profiles for the development of precision medicine strategies for assessing PCa risk. They align with the principles of precision medicine, which emphasize creating approaches that consider an individual’s genetic background, lifestyle, environment, and family health history [64]. Future research could enhance this approach, which has the potential to guide patients in making informed health decisions and reducing healthcare costs by delivering the most effective treatments from the outset.

Thus, given the unique genetic and environmental factors affecting the Puerto Rican population, these biomarkers may enhance personalized treatment strategies aimed to control tumor growth, preventing metastasis, and managing symptoms more effectively. Specifically, N-cadherin expression was identified as a critical determinant of PCa aggressiveness, exhibiting a moderate discriminatory power. This emphasizes N-cadherin’s potential as a classifier for aggressive disease, particularly in Puerto Rican PCa patients. While these findings are encouraging, a prospective study with a larger sample size from Puerto Rican patients is necessary to validate their clinical utility in PCa risk assessment. Additionally, future research should focus on evaluating N-cadherin expression levels alongside other potential biomarkers and comparing these findings across different populations.

While clinicopathological data can provide some insight into a patient’s risk assessment, it lacks precision in predicting individual patient outcomes, making this study significant [57]. Relying solely on clinicopathological data can lead to failure to choose the most effective treatment and potentially harm the patient [57]. Existing PCa prognostic tests, primarily based on genomics, have limitations. They can be expensive, lack clear cost-effectiveness, and often miss the impact of post-translational modifications on gene function [58,59]. Biomarkers have the potential to overcome current prognostic test limitations by aiding tumor stratification while improving cost-effectiveness and patient prognosis. Although the IHC technique used in this study provides a visual assessment of protein expression, it indicates the presence, absence, or relative abundance of proteins. This technique also provides information about the intracellular location of protein, which is crucial for understanding tumor progression [60]. In addition, IHC is generally less expensive, and results can be obtained faster than genomic testing, which requires more complex procedures [61]. Currently, the ProMark test is the only commercially available prognostic test for PCa that relies on protein expression analysis [61]. ProMark is a biopsy-based assay that quantifies eight proteins (CUL2, DERL1, FUS, HSPA9, PDSS2, pS6, SMAD4 and YBX1) demonstrated to be relevant to PCa aggressiveness in men with Gleason Score 3+3 and 3+4 [62,63]. However, the effectiveness of ProMark in predicting PCa aggressiveness in the Puerto Rican population remains uncertain, and further research is needed to evaluate its accuracy in this specific demographic [62].

A limitation of the present study is the use of retrospective biopsy samples, which could be influenced by sample preservation methods, potentially affecting protein stability and IHC staining results. Additionally, obtaining biopsies from the same patient at different time points during disease progression is a significant challenge and may not accurately reflect dynamic changes in biomarker expression over time. Therefore, incorporating a prospective study would minimize the limitations of analyzing heterogeneous samples and facilitate the inclusion of additional patient data, such as MRI results, thus contributing to a more comprehensive understanding of aggressive PCa. Such an approach will enhance the early detection and development of management strategies specifically tailored to the Puerto Rican population. In addition, working with a limited sample size (N=23) was a limitation of our study. While we acknowledge that the small sample size limits the statistical power and complexity of multivariate or machine learning analyses, our goal was to conduct a preliminary study to identify potential biomarkers in a Puerto Rican cohort. These findings serve as a foundation for future prospective studies involving larger cohorts. Ultimately, this study provides valuable insights into the biological behavior of PCa in Puerto Rican men and demonstrates the feasibility of biomarker-based analysis in this population. Thus, we emphasize the need for future studies with a larger Puerto Rican cohort to confirm the observed associations and improve model robustness.

## 5. Conclusions

In conclusion, Phospho-Rb S249, N-cadherin, β-catenin, and E-cadherin are clinically relevant as IHC-based biomarkers for assessing PCa aggressiveness in Puerto Rican men. Specifically, N-cadherin was shown to be a key classifier of tumor aggressiveness, while β-catenin and E-cadherin were associated with tumor size and grade group. Additionally, Phospho-Rb S249 expression was positively correlated with tumor size and the number of positive biopsy cores. Together, the combined expression of these biomarkers may provide a more specific stratification of PCa cases, potentially informing prognosis and therapeutic decision making. As an accessible and cost-effective alternative to genomic testing, IHC biomarkers could support the early identification of high-risk patients and guide personalized treatment strategies, particularly in resource-limited settings. Importantly, this study emphasizes the need for population-specific approaches for PCa management. Puerto Rican men may exhibit unique disease characteristics, and understanding these disparities is crucial for improving healthcare equity and prognostic accuracy across diverse populations. Future large-scale prospective studies are essential to validate these findings and to establish the clinical utility of these biomarkers in routine diagnostic workflows for PCa management.

## Figures and Tables

**Table 1.** Description of PCa tissue characteristics based on Gleason score patterns as defined by the NCCN guidelines.

Gleason patterns	Gleason score	Description
≤3+3	≤6	Well-formed glands
3+4	7	Predominantly well-formed glands with lesser poorly formed/fused/cribriform glands
4+3	7	Predominantly poorly formed/fused/cribriform glands with lesser component of well-formed glands
4+4, 3+5, 5+3	8	Only poorly formed/fused/cribriform glands
4+5, 5+4, 5+5	9 or 10	Lack gland formation (or with necrosis) with or without poorly formed/fused/cribriform glands

**Table 2.** Clinical profile of the Puerto Rican PCa patients after stratifying them by their Gleason scores. Table shows the number of frequency and percentage of contribution in each category as “N(%)”.

Variable	Overall (N=23)	Gleason score ≤ 3 + 4 (N=8)	Gleason score ≥ 4 + 3 (N=15)	*p*-value
**Age (years)**				0.581
Mean (sd)	69.65 (8.10)	67.75 (9.29)	70.67 (7.54)	
Median (p25, p75)	70.0 (66.0, 75.0)	71.5 (64.0, 73.5)	70.0 (66.0, 75.0)	
**Gleason score**				-
6	3 (13.04)	3 (37.50)	0	
7	11 (47.83)	5 (62.50)	6 (40.00)	
8	4 (17.39)	0	4 (26.67)	
9	4 (17.39)	0	4 (26.67)	
10	1 (4.35)	0	1 (6.67)	
**Grade group**				-
1	3 (13.04)	3 (37.50)	0	
2	5 (21.74)	4 (50.00)	1 (6.67)	
3	5 (21.74)	0	5 (33.33)	
4	4 (17.39)	0	4 (26.67)	
5	6 (26.09)	1 (12.50)	5 (33.33)	
**Tumor size (N=22)**				0.142*
Mean (sd)	7.54 (5.60)	4.78 (1.95)	9.11 (6.42)	
Median (p25, p75)	5.8 (4.2, 9.0)	5.25 (3.65, 5.9)	6.75 (4.50, 14.40)	
**Percentage with carcinoma**				0.478*
Mean (sd)	42.65 (29.08)	36.3 (27.71)	46.03 (30.16)	
Median (p25, p75)	34.0 (20.0, 58.0)	30.5 (12.65, 56.5)	34.0 (22.0, 72.0)	
**Number of positive cores**				0.054*
Mean (sd)	5.70 (3.55)	4.00 (3.59)	6.60 (3.29)	
Median (p25, p75)	5.0 (2.0, 8.0)	2.0 (2.0, 6.0)	6.0 (4.0, 8.0)	
**PSA (N=21)**				<0.001*
Mean (sd)	28.81 (38.90)	5.82 (1.04)	40.30 (43.60)	
Median (p25, p75)	11.51 (5.93, 18.74)	5.80 (5.07, 6.62)	16.96 (11.51, 62.25)	

sd: standard deviation, p25 or 75: 25- & 75-percentile,

* Wilcoxon rank test

**Table 3.** Puerto Rican PCa biomarker’s expression after stratifying them by their Gleason scores. Table shows the number of frequency and percentage of contribution in each category as “N(%)”.

Variable	Overall (N=23)	Gleason score ≤ 3 + 4 (N=8)	Gleason score ≥ 4 + 3 (N=15)	*p*-value*
**Phospho-Rb S249**				0.570
Mean (sd)	1.95 (0.47)	1.97 (0.47)	1.93 (0.49)	
Median (p25, p75)	2.0 (1.75, 2.0)	1.75 (1.75, 2.13)	2.0 (1.75, 2.0)	
**N-Cadherin**				0.023
Mean (sd)	0.75 (0.45)	1.06 (0.37)	0.58 (0.41)	
Median (p25, p75)	0.75 (0.25, 1.0)	1.12 (0.75, 1.37)	0.75 (0.25, 1.0)	
**E-cadherin**				0.191
Mean (sd)	1.36 (0.59)	1.56 (0.68)	1.25 (0.53)	
Median (p25, p75)	1.5 (1.0, 2.0)	1.25 (2.12)	1.0 (0.75, 1.75)	
***β*-Catenin**				0.018
Mean (sd)	1.42 (0.48)	1.72 (0.28)	1.27 (0.50)	
Median (p25, p75)	1.5 (1.25, 1.75)	1.75 (1.5, 2.0)	1.25 (1.0, 1.5)	

sd: standard deviation, p25 or 75: 25- & 75-percentile,

* Wilcoxon rank test

**Table 4.** Correlation of Phospho-Rb S249, N-cadherin, β-catenin, and E-cadherin expression with the clinicopathological data of patients with Gleason scores ≤3+4.

Variables	r*	*p*-value
**Phospho-Rb S249**		
Age (years)	0.340	0.410
PSA (ng/ml) (n=7)	0.394	0.382
Grade group	0.125	0.768
Tumor size (mm)	−0.355	0.388
% with carcinoma	−0.051	0.905
Number of positive cores	0.155	0.713
**N-cadherin**		
Age (years)	−0.221	0.599
PSA (ng/ml) (n=7)	−0.367	0.418
Grade group	−0.259	0.536
Tumor size (mm)	0.412	0.310
% with carcinoma	0.449	0.265
Number of positive cores	0.245	0.558
***β*-catenin**		
Age (years)	0.306	0.461
PSA (ng/ml) (n=7)	−0.386	0.393
Grade group	−0.609	0.109
Tumor size (mm)	−0.037	0.931
% with carcinoma	−0.321	0.438
Number of positive cores	−0.421	0.299
**E-cadherin**		
Age (years)	−0.500	0.207
PSA (ng/ml) (n=7)	−0.273	0.554
Grade group	−0.759	0.029
Tumor size (mm)	−0.108	0.798
% with carcinoma	−0.241	0.565
Number of positive cores	−0.449	0.264

* Spearman correlation

**Table 5.** Correlation of Phospho-Rb S249, N-cadherin, β-catenin, and E-cadherin expression with the clinicopathological data in patients with Gleason scores ≥4+3.

Variables	r*	*p*-value
**Phospho-Rb S249**		
Age (years)	−0.300	0.278
PSA (ng/ml) (N=14)	0.120	0.683
Grade group	0.285	0.302
Tumor size (mm) (N=14)	0.616	0.019
% with carcinoma	0.394	0.147
Number of positive cores	0.522	0.046
**N-cadherin**		
Age (years)	0.429	0.111
PSA (ng/ml) (N=14)	0.340	0.234
Grade group	0.061	0.829
Tumor size (mm) (N=14)	−0.208	0.475
% with carcinoma	0.124	0.660
Number of positive cores	0.082	0.771
**β-catenin**		
Age (years)	0.139	0.620
PSA (ng/ml) (N=14)	0.013	0.963
Grade group	−0.069	0.808
Tumor size (mm) (N=14)	0.570	0.033
% with carcinoma	0.635	0.011
Number of positive cores	0.383	0.158
**E-cadherin**		
Age (years)	−0.340	0.215
PSA (ng/ml) (N=14)	−0.357	0.210
Grade group	−0.566	0.028
Tumor size (mm) (N=14)	0.126	0.668
% with carcinoma	0.216	0.439
Number of positive cores	−0.182	0.516

* Spearman correlation

## Data Availability

The data supporting the findings of this study are available on GitHub at https://github.com/svalle19/Puerto-Rico-PCa-data.git.
